# Nationwide Survey of the Surgical Treatment for Hiatal Hernia in Japan

**DOI:** 10.1002/ags3.70079

**Published:** 2025-09-15

**Authors:** Soji Ozawa, Nobuo Omura, Kazuo Koyanagi, Junya Oguma, Akihito Kazuno, Yuko Kitagawa, Yoshihiro Kakeji, Yasushi Toh, Hisahiro Matsubara

**Affiliations:** ^1^ Department of Surgery Tamakyuryo Hospital Machida Japan; ^2^ Tokai University School of Medicine Isehara Japan; ^3^ Department of Surgery National Hospital Organization, Nishisaitama‐Chuo National Hospital Tokorozawa Japan; ^4^ Department of Gastroenterological Surgery Tokai University School of Medicine Isehara Japan; ^5^ Department of Esophageal Surgery National Cancer Center Tokyo Japan; ^6^ Department of Surgery Keio University School of Medicine Tokyo Japan; ^7^ Division of Gastrointestinal Surgery, Department of Surgery Kobe University Graduate School of Medicine Kobe Japan; ^8^ Department of Gastrointestinal Surgery NHO Kyushu Cancer Center Fukuoka Japan; ^9^ Department of Frontier Surgery, Graduate School of Medicine Chiba University Chiba Japan

**Keywords:** fundoplication, hiatal hernia, laparoscopic surgery, paraesophageal hernia, sliding hernia

## Abstract

**Aim:**

This study aimed to clarify patient background characteristics, preoperative findings, surgical factors, and postoperative outcomes according to hernia type in patients who underwent surgery for hiatal hernia in Japan.

**Methods:**

We conducted a retrospective, questionnaire‐based clinical review of patients who underwent surgery between January 2001 and December 2015 at institutions with board‐certified esophagologists accredited by the Japan Esophageal Society. Data from 960 cases across 80 institutions in Japan were analyzed.

**Results:**

Of the 960 cases, data on hernia type were available in 838 and included in the analysis. The distribution was as follows: Type I, 524 cases (63%); Type II, 53 (6%); Type III, 171 (20%); and Type IV, 90 (11%). Compared with Types II–IV, Type I patients were younger, more often male, had longer symptom duration, more heartburn, fewer comorbidities, and more severe esophagitis. Strictures were rare, and surgery was more often indicated due to refractoriness to medical treatment. Type I cases had higher rates of laparoscopic surgery and Toupet fundoplication, with shorter operative times, fewer complications, and shorter hospital stays. They had lower rates of postoperative dysphagia. Risk factors for postoperative dysphagia included Types II–IV hernia (OR 1.676, *p* = 0.002), preoperative dysphagia (OR 1.898, *p* = 0.006), and esophageal strictures (OR 3.102, *p* = 0.016). Hernia type was not associated with postoperative recurrence.

**Conclusion:**

Patients with Type I hernia differed from those with Types II–IV in background characteristics, preoperative findings, surgical factors, and postoperative outcomes. Given the higher risk of postoperative dysphagia in Types II–IV, careful attention to surgical technique is warranted.

## Graphical abstract

This nationwide study of 960 hiatal hernia surgeries in Japan found that patients with Type I hernia had distinct characteristics and more favorable surgical outcomes compared to Types II–IV. Postoperative dysphagia was significantly associated with Types II–IV, preoperative dysphagia, and esophageal strictures, highlighting the need for careful surgical planning in these cases.

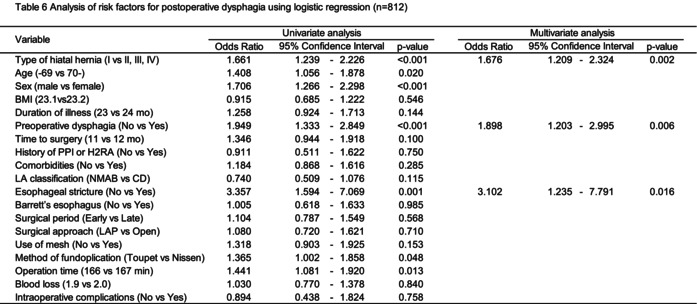

## 1 Introduction

Hiatal hernia is characterized by protrusion of any abdominal structure other than the esophagus into the thoracic cavity through a widening of the hiatus of the diaphragm [1]. Historically, Bowditch was the first to report hiatal hernia in 1853 [2]. Subsequently, in 1926, Akerlund classified esophageal hiatal hernias from the anatomical and radiological perspectives [3], and in 1951, Allison clearly classified them into three types: the sliding type, paraesophageal type, and combined type [4]. Currently, the following classification is widely used [1, 5]: Type I hernias, or sliding hiatal hernias, are characterized by the gastroesophageal junction sliding above the diaphragm. The stomach remains in its usual longitudinal alignment, and the gastric fundus remains below the gastroesophageal junction. Type II hernias, or pure paraesophageal hernias, are characterized by the gastroesophageal junction remaining in its normal anatomical position, but a portion of the gastric fundus herniating through the diaphragmatic hiatus adjacent to the esophagus. Type III hernias, combined types I and II hernias, are characterized by both the gastroesophageal junction and the gastric fundus herniating through the hiatus; the gastric fundus lies above the gastroesophageal junction. Type IV hiatal hernias are characterized by the presence of a structure other than the stomach, such as the omentum, colon, or small bowel, within the hernia sac.

Soresi was the first to report surgical treatment for hiatal hernia in 1919 [6]. His operation consisted of reducing the hernia and closing the opening of the diaphragm. Subsequently, Harrington reported a transabdominal approach in 1928 [7], and in 1952, Sweet et al. reported a transthoracic approach to treating hiatal hernias [8]. In addition to crural repair, Nissen introduced complete circumferential fundoplication in 1956, as an effective anti‐reflux procedure that came to be widely adopted worldwide [9]. However, dysphagia was a common complaint following Nissen fundoplication. To address this issue, surgeons in Europe introduced an anterior partial fundoplication procedure termed Dor fundoplication in 1962 [10], and a 270° posterior partial fundoplication procedure termed Toupet fundoplication in 1963 [11]. In 1991, Dallemagne and Geagea separately reported laparoscopic Nissen fundoplication [12, 13], which led to widespread adoption of the minimally invasive surgery worldwide for the treatment of hiatal hernias. Furthermore, in 1999, Cadière reported robot‐assisted Nissen fundoplication [14], and robot‐assisted anti‐reflux surgery has also been increasingly utilized.

Thus, surgical treatment for hiatal hernia has a history of approximately 100 years, and the aforementioned classification of hiatal hernias into four types is widely accepted. Nonetheless, many aspects of the current status of surgical treatment for hiatal hernia in Japan remain unclear. Therefore, this study was aimed at elucidating the actual conditions of patients with hiatal hernia who were treated by surgery, including the frequency of each type of hernia, the patient background characteristics, the preoperative examination findings, the surgical factors, and the postoperative outcomes. We conducted a nationwide survey of patients with hiatal hernia who had undergone surgery at institutions featuring board‐certified esophagologists by the Japan Esophageal Society on the medical staff. To the best of our knowledge, this study represents the largest‐scale investigation of the clinical aspects of hiatal hernia in Japan, and its findings are expected to provide valuable insights for improved surgical treatment of hiatal hernias in the future in Japan.

## 2 Methods

### 2.1 Study Design and Patients

This study was approved and registered as a research project by the Japan Esophageal Society (No. 2017‐4). A questionnaire‐based, retrospective clinical review was conducted of patients with hiatal hernia who had undergone surgery between January 2001 and December 2015 at institutions featuring board‐certified esophagologists by the Japan Esophageal Society on the medical staff. The patients' data from 960 cases were reviewed based on questionnaire responses collected from 80 institutions (Table S1).

The following data were collected: patient background characteristics (age, sex, body mass index [BMI], duration of illness, symptoms, time to surgery, presence/absence of *Helicobacter pylori* infection, history of medical treatment, comorbidities), examination findings (upper gastrointestinal endoscopy, classification of reflux esophagitis, presence/absence of esophageal stricture and/or Barrett's esophagus, 24‐h pH monitoring, 24‐h impedance‐pH monitoring, esophageal manometry), details of surgery (indication for surgery, surgical approach, use of a mesh, type of mesh used, method of fundoplication adopted, operation time, blood loss, need for blood transfusion, intraoperative complications), and the postoperative course (postoperative hospital stay, postoperative date of resumption of oral intake, duration of postoperative dysphagia, postoperative complications, endoscopic dilatation for postoperative dysphagia, postoperative follow‐up period, improvement of clinical symptoms, postoperative endoscopic findings, occurrence of recurrence, main treatment for recurrence).

Assessment of dysphagia in this study was based on a simple evaluation of the presence or absence of subjective symptoms. Similarly, presence/absence of stricture was determined by the presence or absence of narrowing observed on upper gastrointestinal endoscopy. The definition of postoperative dysphagia in this study encompasses all cases, regardless of severity or duration. Postoperative dysphagia is a symptom that can significantly impair the quality of life. The primary objective of analyzing postoperative dysphagia was to identify the risk factors for its occurrence and to devise surgical strategies that could reduce its incidence. The primary objective of analyzing the recurrence rates was to identify factors that might contribute to recurrence. If any significant factors had been identified, preventive strategies could have been proposed.

We collected individual patient data in accordance with the ethics procedures specified by the Japan Esophageal Society. After the study was approved by the Institutional Review Board (IRB) of Tokai University (approval no. 18R‐171), we submitted the approval notice and an opt‐out consent presentation file (PowerPoint, PPT) to the secretariat of the Japan Esophageal Society. The secretariat uploaded the PPT file to the society's official website and the participating institutions subsequently obtained approval from their respective IRBs. All the procedures were conducted in accordance with the Ethical Guidelines for Medical and Health Research Involving Human Subjects (revised in 2017) issued by the Ministry of Education, Culture, Sports, Science and Technology and the Ministry of Health, Labour and Welfare, Japan [15], which are themselves based on international standards such as the Declaration of Helsinki.

### 2.2 Statistical Analysis

Continuous variables are expressed as medians (interquartile range), and comparisons among the four groups were performed by the Kruskal–Wallis test. If the Kruskal–Wallis test indicated a significant difference, pairwise comparisons between groups were conducted using the Mann–Whitney *U*‐test. Categorical variables are expressed as frequencies (%), and comparisons among the four groups were performed using the chi‐squared test. If the chi‐squared test indicated a significant difference, adjusted standardized residuals were calculated to assess significant differences among specific cells. The Bonferroni method was used to adjust for multiple comparisons. Logistic regression analysis was used to identify factors associated with postoperative dysphagia, and the Cox proportional hazards model was employed to examine factors related to the postoperative recurrence rate. For multivariable analysis, a forward stepwise selection method based on the likelihood ratio was applied. Candidate variables were selected by univariate analysis based on a *p* < 0.10 threshold and incorporated into the final model to consider the clinical significance. Odds ratios or hazard ratios with 95% confidence intervals (95% CI) were calculated to identify statistically significant factors. The postoperative recurrence‐free rates were calculated by the Kaplan–Meier method, and comparisons of the recurrence‐free rates among patients with the four types of hiatal hernia were performed by the log‐rank test. All the statistical analyses were performed using IBM SPSS Statistics for Windows, version 29.0 (IBM Corp., Armonk, NY, USA) and BellCurve for Excel, version 4.08 (Social Survey Research Information Co. Ltd., Tokyo, Japan). A *p*‐value of < 0.05 was set as being indicative of statistical significance.

## 3 Results

### 3.1 Patient Background

Of the data received for 960 cases from 80 institutions, the type of hiatal hernia was documented for 838 cases, which were included in the analysis. The distribution of the hernia types in the study population was as follows: Type I, 524 cases (63%); Type II, 53 cases (6%); Type III, 171 cases (20%); and Type IV: 90 cases (11%). The median age of the study population was 70 years. Patients with Type I were younger than those with Type II, Type III, or Type IV hiatal hernia, and patients with Type II hernias were younger than those with Type IV hernias. The overall male: female distribution was 40%:60%. The proportion of men was higher in the group with Type I hernias, whereas the proportions of women were higher in the groups with Type II or Type III hernias. The median BMI of the overall study population was 23.2 kg/m^2^, while the BMI was higher in patients with Type III than in those with Type IV hernias. The median duration of illness was 24 months, and patients with Type I hernias had a longer disease duration than those with Type IV hernias. In regard to the symptoms, heartburn was the most common symptom, recorded in 45% of all cases. However, it was more frequent in patients with Type I than in those with Type III or Type IV hernias. Chest pain was more frequent in patients with Type II hernias and less frequent in those with Type I hernias. Dysphagia was more common in patients with Type IV hernias, but less common in those with Type I hernias. Cough was less frequent, while reflux was more frequent in the patients with Type I hernias. The median time to surgery was 12 months, and it was shorter in the group with Type IV hernias than in those with Type I or Type III hernias. The proportion of patients without comorbidities was 31% overall, being the highest in patients with Type I hernias and lower in those with Type III or Type IV hernias. Hypertension and cardiac disease were less common in patients with Type I hernias, but more prevalent in patients with Type III hernias (Table 1).

**TABLE 1:** Patient background.

	Type I (*n* = 524)	Type II (*n* = 53)	Type III (*n* = 171)	Type IV (*n* = 90)	Total (*n* = 838)	*p*
1. Age at surgery (years old)	63 [46, 74]	72 [66, 80]	77 [70, 82]	78 [72, 84]	70 [56, 79]	< 0.001, a, b, c, e
2. *Sex*						
Male	273* (52%)	10** (19%)	29** (17%)	25 (28%)	337 (40%)	< 0.001
Female	251** (48%)	43* (81%)	142* (83%)	65 (72%)	501 (60%)	
3. BMI (kg/m^2^)	23.2 [21.0, 25.6]	21.5 [20.4, 25.3]	24.0 [21.0, 26.4]	22.5 [19.1, 25.1]	23.2 [20.8, 25.8]	0.006, f
4. Duration of illness (month)	24 [12, 60]	12 [6, 60]	24 [8, 60]	12 [3, 40]	24 [9, 60]	0.002, c
5. *Symptoms*						
a) Heartburn	298* (57%)	15 (28%)	48** (28%)	14** (16%)	375 (45%)	< 0.001
b) Regurgitation	19 (4%)	0 (0%)	6 (4%)	2 (2%)	27 (3%)	
c) Chest pain	61** (12%)	18* (34%)	31 (18%)	19 (21%)	129 (15%)	
d) Dysphagia	62** (12%)	13 (25%)	37 (22%)	23* (26%)	135 (16%)	
e) Pharyngeal discomfort	28 (5%)	3 (6%)	4 (2%)	2 (2%)	37 (4%)	
f) Cough	22** (4%)	5 (9%)	17 (10%)	7 (8%)	51 (6%)	
g) Hoarseness	12 (2%)	3 (6%)	9 (5%)	3 (3%)	27 (3%)	
h) Asthma‐like attacks	4 (1%)	0 (0%)	3 (2%)	3 (3%)	10 (1%)	
i) Reflux sensation	133* (25%)	7 (13%)	23 (13%)	9 (10%)	172 (21%)	
6. Time to surgery (month)	12 [4, 36]	12 [5, 42]	12 [4, 32]	6 [1, 36]	12 [4, 36]	0.002, c, f
7. *Helicobacter pylori infection*						
a) Yes	25 (5%)	1 (2%)	2 (1%)	2 (2%)	30 (4%)	0.394
b) No	103 (20%)	10 (19%)	26 (15%)	7 (8%)	146 (18%)	
c) No (eradication history)	19 (4%)	3 (6%)	9 (5%)	2 (2%)	33 (4%)	
d) Unknown	368 (71%)	39 (73%)	133 (79%)	77 (88%)	617 (74%)	
8. *History of medical treatment*						
a) Standard dose PPI	257 (49%)	36 (68%)	118 (69%)	46 (51%)	457 (55%)	0.577
b) Double‐dose PPI	25 (5%)	3 (6%)	9 (5%)	2 (2%)	39 (5%)	
c) H2RA	27 (5%)	4 (8%)	11 (6%)	6 (7%)	48 (6%)	
d) Mucosal protectant	36 (7%)	6 (11%)	15 (9%)	5 (6%)	62 (7%)	
e) Prokinetic agent	55 (10%)	7 (13%)	31 (18%)	15 (17%)	108 (13%)	
f) Kampo medicine	8 (2%)	5 (9%)	9 (5%)	2 (2%)	24 (3%)	
9. *Comorbidities*						
a) None	201* (38%)	17 (32%)	31** (18%)	13** (14%)	262 (31%)	< 0.001
b) Hypertension	118** (23%)	14 (26%)	77* (45%)	44 (49%)	253 (30%)	
c) Diabetes	33 (6%)	11 (21%)	18 (11%)	12 (13%)	74 (9%)	
d) Cardiac disease	36** (7%)	8 (15%)	35* (20%)	18 (20%)	97 (12%)	
e) Respiratory disease	40 (8%)	4 (8%)	20 (12%)	15 (17%)	79 (9%)	
f) Liver disease	17 (3%)	1 (2%)	3 (2%)	2 (2%)	23 (3%)	
g) Renal disease	6 (1%)	0 (0%)	5 (3%)	3 (3%)	14 (2%)	
h) Cerebrovascular disease	29 (6%)	5 (9%)	10 (6%)	15 (17%)	59 (7%)	
i) Collagen disease	10 (2%)	4 (8%)	2 (1%)	4 (4%)	20 (2%)	
j) Malignancy	21 (4%)	5 (9%)	10 (6%)	6 (7%)	42 (5%)	

*Note:* a, Type I versus Type II (*p* < 0.05); b, Type I versus Type III (*p* < 0.05); c, Type I versus Type IV (*p* < 0.05); e, Type II versus Type IV (*p* < 0.05); f, Type III versus Type IV (*p* < 0.05).

* A statistically significant cell with a positive adjusted standardized residual.

** A statistically significant cell with a negative adjusted standardized residual.

### 3.2 Examination Findings

Los Angeles classification (LA classification, based on the endoscopic findings) Grade N and Grade M together accounted for 50% of all cases. The proportion of cases with Grade N was lower in the Type I group and higher in the Type II, Type III, and Type IV groups. Patients classified as Grades B, C, and D were more frequent in the Type I group. Esophageal stricture was observed in 4% of all cases, but only rarely in the Type I cases. 24‐h pH monitoring had been conducted in 20% of the patients overall. In this patient group, reflux‐related factors were more frequently identified in the Type I than in the Type III hernia group. The proportion of patients who underwent 24‐h impedance‐pH monitoring was 24% overall, with lower rates in the Type III and Type IV groups as compared with the Type I group. No significant differences were found in the results of 24‐h impedance‐pH monitoring, except in the rates of non‐acidic liquid reflux episodes. Esophageal manometry was performed in 22% of cases overall, with a lower performance rate in the Type III and Type IV hernia groups as compared with the Type I hernia group (Table 2).

**TABLE 2:** Examination findings.

	Type I (*n* = 524)	Type II (*n* = 53)	Type III (*n* = 171)	Type IV (*n* = 90)	Total (*n* = 838)	*p*
**1. Upper gastrointestinal endoscopy**						
1.1. *Classification of reflux esophagitis (Los Angeles classification)*						
N	97** (19%)	26* (49%)	79* (46%)	49* (54%)	251 (30%)	< 0.001
M	104 (20%)	14 (26%)	32 (19%)	21 (23%)	171 (20%)	
A	109 (21%)	7 (13%)	23 (13%)	11 (12%)	150 (18%)	
B	76* (14%)	2 (4%)	13 (8%)	2 (2%)	93 (11%)	
C	69* (13%)	1 (2%)	12 (7%)	2 (2%)	84 (10%)	
D	69* (13%)	3 (6%)	12 (7%)	5 (7%)	89 (11%)	
1.2. *Esophageal stricture*						
Present	13** (2%)	2 (4%)	13 (8%)	6 (7%)	34 (4%)	0.015
Not present	511* (98%)	51 (96%)	158 (92%)	84 (93%)	804 (96%)	
1.3. *Barrett's esophagus*						
a) SSBE	43 (8%)	5 (9%)	14 (8%)	9 (10%)	71 (9%)	0.699
b) LSBE	8 (2%)	0 (0%)	0 (0%)	1 (1%)	9 (1%)	
c) None	473 (90%)	48 (91%)	157 (92%)	80 (89%)	758 (90%)	
Present	51 (10%)	5 (9%)	14 (8%)	10 (11%)	80 (10%)	0.887
Not present	473 (90%)	48 (91%)	157 (92%)	80 (89%)	758 (90%)	
**2. 24‐h pH monitoring**						
Performed	115 (22%)	7 (13%)	33 (19%)	9 (10%)	164 (20%)	0.036
Not performed	405 (78%)	46 (87%)	138 (81%)	80 (90%)	669 (80%)	
2.1. *Findings*						
(a) Number of reflux episodes (/day)	145 [23, 245]	43 [15]	58 [24, 146]	120 [19, 206]	115 [22, 226]	0.296
(b) Time with pH < 4 (%)	16.1 [4.9, 37.2]	2.6 [0.3]	6.6 [1.9, 16.5]	5.3 [2.5, 13.5]	12.5 [3.2, 28.0]	0.005, b
(c) DeMeester score	54.2 [15.3, 140.6]	15.8 [2.4]	7.6 [1.2, 43.5]	—	42.6 [8.3, 101.9]	< 0.001, b
**3. 24‐h impedance‐pH monitoring**						
Performed	171* (35%)	5 (9%)	13** (8%)	3** (4%)	192 (24%)	< 0.001
Not performed	317** (65%)	48 (91%)	156* (92%)	82* (96%)	603 (76%)	
3.1. *Findings*						
(a) Total liquid reflux episodes (/day)	53 [36, 84]	29 [29, 29]	66 [47, 98]	17 [17, 17]	54 [36, 84]	0.141
(b) Acidic liquid reflux episodes (/day)	28 [12, 44]	1 [20, 20]	38 [7, 51]	1 [1, 1]	29 [12, 45]	0.294
(c) Non‐acidic liquid reflux episodes (/day)	25 [14, 39]	9 [9, 9]	41 [30, 62]	16 [16, 16]	26 [15, 41]	0.017, b
(d) Time with pH < 4 (%)	3.8 [0.9, 10.8]	11.7 [11.7, 11.7]	23.6 [3.0, 65.3]	3.1 [3.1, 3.1]	4.1 [1.1, 12.1]	0.056
(e) DeMeester score	11.5 [2.9, 32.2]	33 [33, 33]	8.9 [2.6, 36.9]	ー	11.6 [3.0, 32.8]	0.62
(f) Symptom Index (SI) (%)	78 [50, 100]	ー	100 [78, 100]	ー	80.5 [50, 100]	0.129
(g) Symptom‐association probability (SAP) (%)	100 [94, 100]	ー	95 [73, 100]	ー	100 [94, 100]	0.568
**4. Esophageal manometry**						
Performed	162* (31%)	5 (9%)	10** (6%)	5** (6%)	182 (22%)	< 0.001
Not performed	358** (69%)	48 (91%)	160* (94%)	85* (94%)	651 (78%)	
4.1. *Findings*						
(a) LES pressure (mmHg)	16.6 [10.4, 22.3]	16.5 [16.5, 16.5]	11.3 [7.3, 18.3]	34.5 [30.8, −]	16.5 [10.4, 22.4]	0.104
(b) LES length (cm)	2.9 [2.2, 4.0]	1.7 [1.7, 1.7]	2.8 [2.1, 3.5]	1.5 [1.0, −]	2.9 [2.1, 3,9]	0.131

*Note:* b, Type I versus Type III (*p* < 0.05).

* A statistically significant cell with a positive adjusted standardized residual.

** A statistically significant cell with a negative adjusted standardized residual.

### 3.3 Details of Surgery

The most common surgical indication was refractoriness to medical treatment, accounting for 57% of all cases. This indication was the most frequent in the Type I group, and less frequent in the Type III and Type IV groups. Extraesophageal symptoms were less common in the Type I group, but more frequent in the Type II and Type IV groups. The surgical approach was laparoscopic in 85% of cases overall, with this approach being most frequent in the Type I cases and less frequent in the Type III and Type IV groups. Conversely, open surgery was less common in the Type I group and more prevalent in the Type III and Type IV groups. The overall mesh usage rate was 16%, with a lower rate in the Type I group and higher rates in the Type III and Type IV groups. The most commonly used type of mesh was a polyester mesh, which accounted for 44% of all cases. Its usage rate was lower for the Type I cases and higher for the Type III cases. The second most commonly used type of mesh, the expanded polytetrafluoroethylene type of mesh (ePTFE), was used more frequently for Type I cases and less frequently used for the Type III and Type IV cases. Among fundoplication methods, Toupet fundoplication was the most commonly adopted (59%), with a higher rate of its adoption for Type I cases than for Type II cases. The median operation time was 167 min, with a shorter duration of operation in the Type I group as compared with the Type II, Type III, and Type IV groups. The median blood loss was 0 mL, with the blood loss being lower in the Type I group than in the Type II, Type III, and Type IV groups. None of the cases in the Type I group required blood transfusions. The overall intraoperative complication rate was 5%; it was lower in the Type I group and higher in the Type III group. The most common intraoperative complications were pleural injury, esophageal perforation, and injury to the anterior trunk of the vagus nerve, in that order (Table 3).

**TABLE 3:** Details of surgery.

	Type I (*n* = 524)	Type II (*n* = 53)	Type III (*n* = 171)	TypeIV (*n* = 90)	Total (*n* = 838)	*p*
1. *Indications for surgery*						
Refractoriness to medical treatment	322* (68%)	23 (39%)	82** (46%)	25** (31%)	452 (57%)	< 0.001
Age, treatment duration, QOL, medical costs, etc.	69 (15%)	10 (17%)	38 (21%)	16 (20%)	133 (17%)	
Presence of strictures or Barrett's esophagus	18 (4%)	2 (3%)	7 (4%)	2 (2%)	29 (4%)	
Extraesophageal symptoms such as asthma, hoarseness, cough, chest pain, or aspiration	64** (13%)	24* (41%)	52 (29%)	38* (47%)	178 (22%)	
2. *Surgical approach*						
a) Laparoscopic	474* (91%)	42 (79%)	130** (77%)	61** (68%)	707 (85%)	< 0.001
b) Open abdominal	43** (8%)	11 (21%)	36* (21%)	28* (31%)	118 (14%)	
c) Other	5 (1%)	0 (0%)	3 (2%)	1 (1%)	9 (1%)	
3. *Use of mesh*						
Yes	50** (10%)	7 (13%)	46* (27%)	34* (38%)	137 (16%)	< 0.001
No	471* (90%)	46 (87%)	124** (73%)	56** (62%)	697 (84%)	
4. *Type of mesh*						
Polypropylene	2 (4%)	2 (29%)	3 (7%)	6 (17%)	13 (10%)	< 0.001
Polyester	10** (20%)	3 (43%)	30* (65%)	17 (50%)	60 (44%)	
Expanded polytetrafluoroethylene	31* (62%)	0 (0%)	4** (9%)	1** (3%)	36 (26%)	
Polypropylene + expanded polytetrafluoroethylene	2 (4%)	1 (14%)	2 (4%)	4 (12%)	9 (7%)	
Polyglactin	0 (0%)	1 (14%)	1 (2%)	0 (0%)	2 (1%)	
Others	5 (10%)	0 (0%)	6 (13%)	6 (18%)	17 (12%)	
5. *Method of fundoplication*						
a) Nissen	167 (32%)	27 (52%)	62 (37%)	17 (21%)	273 (33%)	< 0.001
b) Toupet	331* (64%)	16** (31%)	91 (54%)	43 (54%)	481 (59%)	
c) Dor	4** (1%)	4 (8%)	7 (4%)	5 (6%)	20 (2%)	
d) Other	17** (3%)	5 (9%)	8 (5%)	15* (19%)	45 (6%)	
6. Operation time (min)	152 [115, 201]	196 [122, 260]	198 [160, 252]	206 [150, 297]	167 [121, 230]	< 0.001, a, b, c
7. Blood loss (mL)	0 [0, 5]	5 [0, 69]	10 [0, 60]	30 [0, 150]	0 [0, 25]	< 0.001, a, b, c, f
8. *Blood transfusion*						
Yes	0** (0%)	1 (2%)	2 (1%)	2 (2%)	5 (1%)	0.022
None	523* (100%)	51 (98%)	169 (99%)	88 (98%)	831 (99%)	
9. *Intraoperative complications*						
Yes	15** (3%)	0 (0%)	16* (9%)	6 (9%)	37 (5%)	< 0.001
None	508* (97%)	53 (100%)	155** (91%)	64 (91%)	780 (95%)	
a) Injury to anterior vagal trunk	1 (7%)	0 (0%)	2 (13%)	2 (33%)	5 (14%)	
b) Injury to posterior vagal trunk	2 (13%)	0 (0%)	0 (0%)	1 (17%)	3 (8%)	
c) Pleural injury	2 (13%)	0 (0%)	8 (50%)	1 (17%)	11 (30%)	
d) Pneumothorax	1 (7%)	0 (0%)	0 (0%)	0 (0%)	1 (3%)	
e) Splenic injury	3 (20%)	0 (0%)	0 (0%)	0 (0%)	3 (8%)	
f) Injury to diaphragm crura	1 (7%)	0 (0%)	0 (0%)	0 (0%)	1 (3%)	
g) Esophageal perforation	4 (27%)	0 (0%)	0 (0%)	2 (33%)	6 (16%)	
h) Gastric perforation	1 (7%)	0 (0%)	2 (13%)	0 (0%)	3 (8%)	
i) Other	0 (0%)	0 (0%)	4 (25%)	0 (0%)	4 (11%)	

*Note:* a, Type I versus Type II (*p* < 0.05); b, Type I versus Type III (*p* < 0.05); c, Type I versus Type IV (*p* < 0.05); f, Type III versus Type IV (*p* < 0.05).

* A statistically significant cell with a positive adjusted standardized residual.

** A statistically significant cell with a negative adjusted standardized residual.

### 3.4 Postoperative Course

The median postoperative hospital stay was 8 days overall. It was shorter in patients with Type I hernias as compared to those with Type III and Type IV hernias and also shorter in Type II hernia patients as compared with Type IV hernia patients. The shortest time to resumption of oral intake was observed in the patients with Type I hernias, while it was longer in patients with Type III and Type IV hernias. The proportion of patients without any postoperative dysphagia was 63% overall, with the highest rate of such patients in the Type I group. The dysphagia resolved within a month in 20% of patients, within 1–3 months in 11% of patients, and persisted for more than 3 months in 6% of patients. The proportion of patients without postoperative complications was 84%, with the highest rate of patients without postoperative complications in the Type I group. The median postoperative follow‐up period was 24 months, being longer in the Type I groups as compared with the Type IV group. The endoscopic findings of reflux esophagitis improved in 75% of all cases, with the greatest rate of improvement in the Type I group and lowest rates of improvement in the Type II group (Table 4).

**TABLE 4:** Postoperative course.

	Type I (*n* = 524)	Type II (*n* = 53)	Type III (*n* = 171)	TypeIV (*n* = 90)	Total (*n* = 838)	*p*
1. Postoperative hospital stay (day)	7 [7, 10]	8 [6, 15]	10 [7, 15]	11 [8, 18]	8 [7, 12]	< 0.001, b,c,e
2. Postoperative date of resumption of oral intake	2 [2, 2]	2 [2, 3]	2 [2, 4]	3 [2, 6]	2 [2, 3]	< 0.001, a,b,c,e,f
3. *Duration of postoperative dysphagia*						
a) None	343* (68%)	29 (56%)	95 (56%)	46 (55%)	513 (63%)	0.018
b) Within 1 month	80** (16%)	13 (25%)	42 (25%)	24 (29%)	159 (20%)	
c) Within 3 months	61 (12%)	7 (13%)	18 (11%)	8 (9%)	94 (11%)	
d) Over 3 months	23 (4%)	3 (6%)	14 (8%)	6 (7%)	46 (6%)	
4. *Postoperative complications*						
a) None	457* (88%)	46 (85%)	132 (77%)	66 (73%)	701 (84%)	0.001
b) Bleeding	0 (0%)	0 (0%)	0 (0%)	1 (1%)	1 (0%)	
c) Infections (e.g., pneumonia)	5 (1%)	0 (0%)	6 (3%)	3 (4%)	14 (2%)	
d) Persistent dysphagia	33 (7%)	5 (9%)	13 (8%)	9 (10%)	60 (7%)	
e) Gas‐bloat syndrome (difficulty belching)	7 (1%)	1 (2%)	5 (3%)	2 (2%)	15 (2%)	
f) Other	16** (3%)	2 (4%)	16 (9%)	9 (10%)	43 (5%)	
5. *Endoscopic dilation for postoperative dysphagia*						
Yes	10 (6%)	1 (4%)	7 (9%)	3 (8%)	21 (7%)	0.757
No	154 (94%)	22 (96%)	67 (91%)	35 (92%)	278 (93%)	
6. Postoperative follow‐up period (month)	25 [11, 51]	24 [6, 51]	24 [8, 45]	19 [5, 37]	24 [9, 49]	0.037, c
7. *Improvement of clinical symptoms*						
a) Yes	255 (79%)	41 (80%)	123 (75%)	56 (67%)	475 (76%)	0.109
b) Moderate	59 (18%)	7 (14%)	39 (24%)	23 (28%)	128 (21%)	
c) No	9 (3%)	3 (6%)	2 (1%)	4 (5%)	18 (3%)	
8. *Postoperative endoscopic findings (reflux esophagitis)*						
a) Improved	211* (84%)	9** (39%)	47 (66%)	25 (59%)	292 (75%)	< 0.001
b) No change	37** (15%)	12* (52%)	20 (28%)	15 (36%)	84 (22%)	
c) Worsened	4 (1%)	2 (9%)	4 (6%)	2 (5%)	12 (3%)	
9. *Recurrence*						
a) No	472 (92%)	46 (90%)	154 (93%)	80 (91%)	752 (92%)	0.857
b) Yes	41 (8%)	5 (10%)	11 (7%)	8 (9%)	65 (8%)	
10. *Main treatment for recurrence*						
a) Observation	21 (49%)	3 (50%)	3 (23%)	4 (50%)	31 (44%)	0.085
b) Medication	18 (42%)	0 (0%)	6 (46%)	2 (25%)	26 (37%)	
c) Reoperation	4 (9%)	3 (50%)	4 (31%)	2 (25%)	13 (19%)	

*Note:* a, Type I versus Type II (*p* < 0.05); b, Type I versus Type III (*p* < 0.05); c, Type I versus Type IV (*p* < 0.05); e, Type II versus Type IV (*p* < 0.05); f, Type III versus Type IV (*p* < 0.05).

* A statistically significant cell with a positive adjusted standardized residual.

** A statistically significant cell with a negative adjusted standardized residual.

### 3.5 Additional Herniated Organs in Patients With Type IV Hiatal Hernias

In the patients with Type IV hiatal hernias, organs other than the stomach that were most frequently herniated included the transverse colon (61%), followed by the small intestine and the omentum (Table 5).

**TABLE 5:** Additional herniated organs in patients with Type IV hiatal hernia.

Organs	*n*	%
Transverse colon	63	61%
Small intestine	14	13%
Omentum	13	12%
Duodenum	8	8%
Pancreas	5	5%
Spleen	1	1%
Total	104	100%

### 3.6 Risk Factors for Postoperative Dysphagia

Logistic regression analysis identified Type II, Type III, and Type IV hernias (vs. Type I hernias), preoperative dysphagia (vs. its absence), and preoperative endoscopic stenosis (vs. its absence) as significant factors for postoperative dysphagia (Table 6).

**TABLE 6:** Analysis of risk factors for postoperative dysphagia using logistic regression (*n* = 812).

Variable	Univariate analysis			Multivariate analysis		
	Odds ratio	95% confidence interval	*p*	Odds ratio	95% confidence interval	*p*
Type of hiatal hernia (I vs. II, III, IV)	1.661	1.239–2.226	< 0.001	1.676	1.209–2.324	0.002
Age (≤ 69 vs. ≥ 70 years)	1.408	1.056–1.878	0.020			
Sex (male vs. female)	1.706	1.266–2.298	< 0.001			
BMI (≤ 23.1 vs. ≥ 23.2 kg/m^2^)	0.915	0.685–1.222	0.546			
Duration of illness (≤ 23 vs. ≥ 24 months)	1.258	0.924–1.713	0.144			
Preoperative dysphagia (no vs. yes)	1.949	1.333–2.849	< 0.001	1.898	1.203–2.995	0.006
Time to surgery (≤ 11 vs. ≥ 12 months)	1.346	0.944–1.918	0.100			
History of PPI or H2RA (no vs. yes)	0.911	0.511–1.622	0.750			
Comorbidities (no vs. yes)	1.184	0.868–1.616	0.285			
LA classification (NMAB vs. CD)	0.74	0.509–1.076	0.115			
Esophageal stricture (no vs. yes)	3.357	1.594–7.069	0.001	3.102	1.235–7.791	0.016
Barrett's esophagus (no VS. yes)	1.005	0.618–1.633	0.985			
Surgical period (early vs. late)	1.104	0.787–1.549	0.568			
Surgical approach (lap vs. open)	1.08	0.72–1.621	0.710			
Use of mesh (no vs. yes)	1.318	0.903–1.925	0.153			
Method of fundoplication (Toupet vs. Nissen)	1.365	1.002–1.858	0.048			
Operation time (≤ 166 vs. ≥ 167 minutes)	1.441	1.081–1.92	0.013			
Blood loss (≤ 1.9 vs. ≥ 2.0 mL)	1.03	0.77–1.378	0.840			
Intraoperative complications (no vs. yes)	0.894	0.438–1.824	0.758			

### 3.7 Recurrence‐Free Survival

**FIGURE 1:**
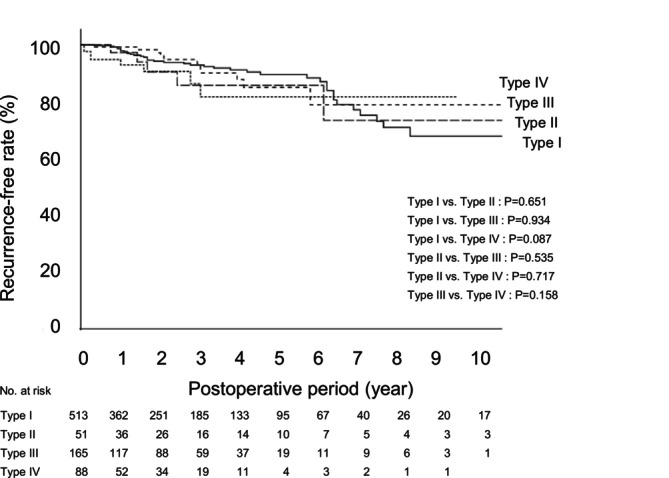
The postoperative recurrence‐free survival was calculated using the Kaplan–Meier method, and comparisons between recurrence‐free survival curves by type were performed using the log‐rank test.

The analysis of postoperative recurrence‐free survival using the Cox proportional hazards model was performed with 21 variables, which included the 19 variables listed in Table 6, along with postoperative dysphagia and postoperative complications. No significant variables were identified (*p* < 0.05), although a trend was observed for comorbidities (*p* = 0.066). Calculation and comparison of the postoperative recurrence‐free survival by the hernia type using the Kaplan–Meier method and log‐rank test revealed no significant differences among the hernia types. The 5‐year recurrence‐free rate was 89.5% in Type I cases, 85.7% in Type II cases, 85.0% in Type III cases, and 81.7% in Type IV cases (Figure 1).

### 3.8 Temporal Trends in Surgical Procedures

The proportion of laparoscopic surgeries remained around 80%, but increased to 89% in the final period (2013–2015). In relation to the method used for fundoplication, complete Nissen fundoplication was adopted in 56% of cases in 2001–2003, which declined to below 40% after 2004. Cases from 2001 to 2003 accounted for only 8.3% of the total (Table 7). Univariate analysis conducted after dividing the cohort into two groups, namely, early (first 7 years 6 months) and late (second 7 years 6 months), showed no significant difference in the incidence of postoperative dysphagia between the two groups (*p* = 0.568, Table 6). Similarly, the analysis also identified no significant difference in the incidence of postoperative recurrence between the two groups (*p* = 0.831).

**TABLE 7:** Temporal trends in surgical approach and method of fundoplication.

Period (year)	2001–2003	2004–2006	2007–2009	2010–2012	2013–2015	Total	*p*
*Surgical approach*							
Laparoscopic	53 (77%)	82 (82%)	79** (75%)	173 (86%)	320* (89%)	707 (85%)	0.003
Open abdominal	16 (23%)	15 (15%)	25* (24%)	27 (13%)	35** (10%)	118 (14%)	
Other	0 (0%)	3 (3%)	1 (1%)	2 (1%)	3 (1%)	9 (1%)	
Total	69 (100%)	100 (100%)	105 (100%)	202 (100%)	358 (100%)	834 (100%)	
*Method of fundoplication*							
Nissen	38* (56%)	34 (34%)	40 (40%)	69 (35%)	92** (26%)	273 (33%)	< 0.001
Toupet	22** (32%)	60 (60%)	48 (48%)	120 (61%)	231* (66%)	481 (59%)	
Dor	1 (1%)	4 (4%)	4 (4%)	1 (1%)	10 (3%)	20 (2%)	
Other	7 (10%)	2 (2%)	9 (9%)	8 (4%)	19 (5%)	45 (5%)	
Total	68 (100%)	100 (100%)	101 (100%)	198 (100%)	352 (100%)	819 (100%)	

* A statistically significant cell with a positive adjusted standardized residual.

** A statistically significant cell with a negative adjusted standardized residual.

## 4 Discussion

In this study, we analyzed the data of patients with esophageal hiatal hernias who had undergone surgical treatment in Japan by the hernia type. The classification into the four types of hiatal hernia is based on the concept illustrated by Allison in 1948 [4], which was subsequently refined to the classification used today [5]. Accurate determination of the hiatal hernia type in patients with Type II hiatal hernias is difficult. In 2023, Ceron et al. conducted a detailed analysis of 846 cases of paraesophageal hernias and found no cases of Type II hernias, which led them to suggest that Type II hiatal hernias, as currently defined, may actually not exist at all [16]. Clinically, given the difficulty in precisely diagnosing the hernia type in patients with Type II hernias both preoperatively and intraoperatively, it is practical from the point of view of the treatment strategy to be adopted, to consider Type II, Type III, and Type IV together, as proposed in the 2024 SAGES guideline [17].

The distribution of the hiatal hernia types among the surgical cases was as follows: Type I, 63%; Type II, 6%; Type III, 20%; and Type IV, 11%. According to an analysis of 8904 cases by Fuchs et al. the distribution was as follows: Type I, 84%; Type II, 4%; Type III, 11%; and Type IV: 1% [18]. While both studies found that Type I hernias were the most common and Type III hernias were the second most common, the proportion of Type I cases was higher in Fuchs' report than in our survey. This difference could possibly be attributable to the higher proportion of Western patients in Fuchs' study as compared to the Japanese patient population in this study.

The relationship between hiatal hernia, age, and sex was previously investigated by Furukawa et al. in a study of 6010 Japanese patients undergoing endoscopy [19]. They found that as compared with individuals aged 30–39 years, women over the age of 60 years and men over the age of 80 years old showed significantly higher prevalences of hiatal hernia. In addition, Abdelmoaty et al. reported that hiatal hernias tend to enlarge over time and that 25% of sliding hiatal hernias eventually become paraesophageal hernias [20]. Considering these findings, it is reasonable to conceive why patients with a smaller hernia opening, as in those with Type I hernias, were younger than those with larger hernia openings, as in those with Type II, Type III, and Type IV hernias. Furthermore, the higher proportion of women in the patient groups with the more advanced Type III and Type IV hernias may also be explained by these findings.

As for the symptoms, heartburn was more common in the Type I hernia group, likely due to the involvement of gastroesophageal reflux. In contrast, dysphagia was more common in the Type IV hernia group, possibly due to gastric volvulus or significant angulation of the lower esophagus. The longer disease duration and time to surgery in the Type I hernia group as compared with the Type IV hernia group could be attributable to the fact that heartburn in patients with Type I hernias can be managed to some extent with antisecretory agents, whereas the obstructive symptoms in patients with Type IV hernias are not responsive to medical treatment, necessitating earlier surgical intervention. The association between comorbidities and the development of hiatal hernia appears to be minimal. The higher prevalence of comorbidities in the Type III and Type IV hernia groups is likely due to the older age of these patients.

The higher proportion of patients with Grade B, Grade C, or Grade D according to the LA classification (based on the endoscopic findings) in the Type I hernia group suggests that gastroesophageal reflux (GER), and therefore, reflux esophagitis, is more frequent in this group. In contrast, the higher proportion of patients classified into LA classification Grade N in the Type II, Type III, and Type IV hernia groups indicated that GER was less common in patients with these hernia types. Esophageal stricture was rarely observed in the Type I hernia group, likely because the lumen at the gastroesophageal junction remains relatively open. However, in Type II, Type III, and Type IV hernias, the degree of gastric displacement into the mediastinum may result in significant angulation between the esophagus and stomach, leading to a higher frequency of esophageal stricture findings.

The lower rates of 24‐h pH monitoring, 24‐h impedance‐pH monitoring, and esophageal manometry in the Type III and Type IV hernia groups as compared with the Type I hernia group could be attributable to the difficulty in inserting the transnasal probes into a stomach that has herniated into the mediastinum. While the overall performance rate of these tests was only 20%–24%, which made any meaningful comparisons among the different hernia types difficult, 24‐h pH monitoring confirmed that GER was more frequent in the Type I hernia group than in the Type III hernia group. Clinically, given the difficulty in performing the tests for GER (24‐h pH monitoring, 24‐h impedance‐pH monitoring, and esophageal manometry) in the patients with Type II, Type III, and Type IV hernias, it might be reasonable to omit these tests in these cases.

The higher proportion of refractoriness to medical treatment as an indication for surgery in the Type I hernia group suggests that medical therapy is often attempted before considering surgery. In contrast, this factor was a less frequent indication for surgery in the Type III and Type IV hernia groups, for which medical treatment is known to be less effective. Conversely, extraesophageal symptoms, such as asthma, hoarseness, cough, chest pain, and aspiration were more common in patients with Type II and Type IV hernias, likely due to oral regurgitation caused by mechanical stenosis.

Regarding the surgical approach, laparoscopic surgery was more commonly performed in the Type I hernia group as compared with Type III and Type IV hernia groups. This is likely because Type I hernias involve a smaller volume of herniated organs in the mediastinum, making dissection easier and reducing the complexity of the repair. In contrast, open surgery was more frequently required for patients with Type III and Type IV hernias due to the larger hernia sacs, and therefore, greater difficulty of dissection. The frequency of use of mesh reinforcement was lower in the Type I hernia group and higher in the Type III and Type IV hernia groups, reflecting the proportional relationship between the size of the hernia defect and the likelihood of mesh placement. Although multiple mesh types were used, the selection appeared to be influenced by individual surgeons' preferences, with the polyester mesh being the most frequently used.

In regard to the type of fundoplication, Toupet fundoplication was performed in approximately 60% of cases, with a particularly high rate in the Type I hernia group. This may reflect a preference shown by surgeons to minimize the risk of postoperative dysphagia. Surgical procedures are generally easier in Type I cases due to the smaller hernia defects and fewer herniated organs, resulting in shorter operative times and lower blood loss, as compared to the case for patients with Type II, Type III, or Type IV hernias. This may also explain the lower incidence of intraoperative complications in patients with Type I as compared with Type III hernias.

The shorter postoperative hospital stay and earlier resumption of oral intake in the Type I hernia group as compared with the Type III and Type IV hernia groups likely reflects the lower invasiveness of surgery for Type I hernias. Postoperative dysphagia was observed in 37% of cases, although this percentage improved over time. This could be attributed to resolution of the inflammation at the gastroesophageal junction and patients gradually adapting their eating habits to their condition, such as taking smaller bites and eating more slowly. The proportion of patients with no postoperative complications was 84%, with the highest rate of patients without postoperative complications in the Type I group, likely due to the lower complexity of surgery and less invasive nature of the surgery in this group. The most significant improvement of the endoscopic findings of reflux esophagitis was observed in the patients with Type I hernias. This could be attributable to the underlying pathophysiology of Type I hernias, which are inherently more susceptible to gastroesophageal reflux, thereby allowing the antireflux mechanism of fundoplication to exert its effect more effectively.

Among the additional herniated organs in patients with Type IV hiatal hernias, the transverse colon was the most frequently involved, followed by the small intestine and omentum; this finding could be explained by these organs being anatomically close to the esophageal hiatus and having greater mobility within the abdominal cavity.

Logistic regression analysis to identify factors associated with postoperative dysphagia identified Type II, Type III, and Type IV hernia, preoperative dysphagia, and endoscopic finding of preoperative stenosis as showing significant associations. As the latter two factors were associated with the hernia type, the hernia type may itself serve as a useful predictor of the occurrence of postoperative dysphagia. A similar analysis for dysphagia persisting for more than 3 months also identified preoperative dysphagia as a significant factor (data not shown), indicating the importance of these variables.

We did not identify any factor that was significantly associated with the postoperative recurrence. Although it was considered that there could be some influence of comorbidities, we could not identify any statistically significant association. In regard to the postoperative recurrence‐free survival, there were no significant differences among the patients with the four hernia types. The 5‐year recurrence‐free rate was in the 80% range, indicating that the hernia type has no influence on the long‐term outcomes.

To address the issue that the cohort includes cases from a wide time range, we have added Table 7 which summarizes the distribution (in 3‐year intervals) of the surgical approach adopted and method of fundoplication used over the 15‐year time period. Laparoscopic surgeries accounted for approximately 80% throughout the study period. Complete Nissen fundoplication was performed in less than 40% of cases after 2004. As cases from 2001 to 2003 comprised only 8.3% of the total, their influence on the analytical results was regarded as minimal. Univariate analysis comparing early and late periods (first and second 7.5 years) revealed no significant differences in postoperative dysphagia or recurrence. Therefore, we considered that the surgical outcomes were unaffected by the period of time in which the surgery was performed.

Our identification of Types II–IV hernias, preoperative dysphagia, and esophageal strictures as significant risk factors for postoperative dysphagia is considered to be of particular clinical significance. Based on the findings, we recommend that in patients at risk, surgical techniques, such as a loose crural repair or partial (non‐circumferential) fundoplication should be considered to prevent obstruction from the distal esophagus to the esophagogastric junction. Intraoperative endoscopic confirmation that the lumen is adequately secured, by ensuring anterior visibility through insufflation, may also be a useful strategy.

There are several limitations of this study. Firstly, this study did not obtain detailed information on the specific diagnostic methods employed to determine hernia type in each case. Therefore, it is assumed that classification was based on a comprehensive evaluation of available preoperative diagnostic modalities—such as endoscopy, computed tomography, and fluoroscopy—along with intraoperative findings. The possibility of inter‐institutional variation in classification criteria is considered minimal, as all data were derived from institutions staffed by board‐certified esophagologists accredited by the Japan Esophageal Society, where a uniformly high level of expertise in esophageal diseases can reasonably be assumed. Determination of the type of hiatal hernia, especially of Type II hernias, is difficult; therefore, if we had created and used clear diagnostic criteria to determine the type of hernia, the results might have been different.

Secondly, we did not investigate the size of the enlarged hernia orifice, that is, the size of the hiatal hernia. Data on this variable would have been useful for interpreting the results by type. As the human population ages, it is expected that the number of patients with Type II, Type III, and Type IV hiatal hernias will increase, and it will be necessary to accumulate more cases to analyze the actual conditions of patients with these three types of hiatal hernias.

Thirdly, in the present study, dysphagia was assessed using a simple evaluation based on the presence or absence of subjective symptoms, and stricture was similarly identified by the presence of luminal narrowing observed on upper gastrointestinal endoscopy. For a more detailed and objective analysis, the use of validated scoring systems to quantify the severity of dysphagia and stricture may be warranted.

Fourthly, as the study was based on retrospective questionnaire responses, the completeness and reliability of data, particularly for older cases, may have been limited. To facilitate future prospective studies, the use of standardized nationwide databases such as the National Clinical Database (NCD) [21] may allow for more accurate, comprehensive, and longitudinal data collection, thereby improving the quality and generalizability of the findings.

In conclusion, this study showed that patients with Type I hernia differed from those with Type II, III, and IV hernias in terms of background characteristics, preoperative findings, surgical factors, and postoperative outcomes. As Type II, III, and IV hernias are more likely to cause postoperative dysphagia, careful attention should be paid to the surgical technique adopted.

## Author Contributions

**Soji Ozawa:** conceptualization, writing – original draft, formal analysis, methodology, data curation, project administration, writing – review and editing, investigation, visualization. **Nobuo Omura:** conceptualization, writing – review and editing, methodology. **Kazuo Koyanagi:** conceptualization, methodology, writing – review and editing. **Junya Oguma:** conceptualization, writing – review and editing. **Akihito Kazuno:** conceptualization, writing – review and editing. **Yuko Kitagawa:** conceptualization, writing – review and editing. **Yoshihiro Kakeji:** conceptualization, writing – review and editing. **Yasushi Toh:** conceptualization, writing – review and editing. **Hisahiro Matsubara:** conceptualization, writing – review and editing.

## Ethics Statement

This study was approved and registered by the Japan Esophageal Society (no. 2017‐4). After the study was approved by the institutional review board (IRB) of Tokai University (approval no. 18R‐171), the Japan Esophageal Society uploaded the opt‐out consent information, and the participating institutions subsequently obtained approval from their respective IRBs. All the procedures were conducted in accordance with the Ethical Guidelines for Medical and Health Research Involving Human Subjects issued by the Ministry of Education, Culture, Sports, Science and Technology and the Ministry of Health, Labour and Welfare, Japan, which are themselves based on international standards such as the Declaration of Helsinki.

## Conflicts of Interest

Yuko Kitagawa is the editor‐in‐chief of *the Annals of Gastroenterological Surgery*. Yoshihiro Kakeji and Hisahiro Matsubara are members of the editorial board of *the Annals of Gastroenterological Surgery*. All other authors declare no conflicts of interest.

## Supporting information


**Table S1:** Participating institutions.
